# Effectiveness of acupuncture combined with medication for mild cognitive impairment: a systematic review and meta-analysis protocol

**DOI:** 10.1186/s13643-026-03306-7

**Published:** 2026-08-22

**Authors:** Sihao Chen, Haihua Xie, Jiaxing Zhu, Qingxia Li, Weiai Liu, Ting Zhu, ShuLin Xiong, Mi Liu

**Affiliations:** 1 Hunan University of Chinese Medicine, Changsha, 410208 China; 2 The Second Affiliated Hospital of Hunan University of Chinese Medicine, Changsha, Hunan 410005 China

**Keywords:** Acupuncture, Pharmacotherapy, Mild cognitive impairment, Meta-analysis, Safety

## Abstract

**Introduction:**

Cognitive impairment is a global health crisis, which reduces quality of life and adds to the socio-economic burden. Previous meta-analyses have shown that the combination of acupuncture and conventional Western medicine may offer advantages over drug therapy alone. However, uncertainties remain regarding the scope and timeliness of the evidence search, the use of outdated risk-of-bias assessment tools, safety and long-term outcomes, and specific clinical value. Therefore, this study will conduct an updated systematic review and meta-analysis aimed at evaluating the clinical efficacy of acupuncture combined with medication for patients with mild cognitive impairment (MCI), with the hope of providing guidance for optimizing clinical decision-making.

**Methods and analysis:**

Search 9 databases (PubMed, Embase, Cochrane Library, Web of Science, China National Knowledge Infrastructure, Wanfang Data, VIP, China Biology Medicine disc, Scopus) from the start date to July 2026. We will also search trial registries and gray literature sources. Include randomized controlled trials comparing acupuncture combined with medication versus medication alone in patients with MCI. The primary outcomes will be the Montreal Cognitive Assessment Scale (MoCA) and the Mini-Mental State Examination (MMSE). Secondary outcomes included the Modified Barthel Index (MBI), Rey Auditory Verbal Learning Test (RAVLT), Digit Span Test, and Adverse Events (AE). Two researchers will independently conduct selection, extraction, and RoB 2 bias risk assessment. The GRADE framework will be used to assess the quality of evidence. Weighted mean differences (MD) and standardized mean differences (SMD) will be used for continuous outcomes. Relative risks (RRs)/odds ratios (ORs) with 95% confidence intervals (CIs) are going to be used for categorical data. Heterogeneity sources will be explored through sensitivity analysis and subgroup analysis. The publication bias will be assessed through funnel plots and Egger’s test.

**Discussion:**

Comprehensive evidence to optimize clinical decision-making and treatment regarding the efficacy of acupuncture and pharmacotherapy in cognitive impairment.

**Systematic review registration:**

PROSPERO CRD420261367845

**Supplementary Information:**

The online version contains supplementary material available at https://doi.org/10.1186/s13643-026-03306-7.

## Introduction

Mild cognitive impairment (MCI) is the transitional stage between normal aging and dementia, with defects in memory, language, and other aspects [1, 2]. Most activities of daily living (ADL) remain good; MCI is often missed, thus hindering timely intervention [3, 4]. In clinical cohorts, MCI progresses to dementia at an estimated annual rate of approximately 10.9%, creating a substantial burden [5]. There are more than 55 million dementia patients worldwide, and the relevant costs exceed 1 trillion [6]. The cognitive process of MCI is not a strictly unidirectional process. Multidomain lifestyle interventions may help slow cognitive decline in at-risk older adults, highlighting the need for early strategies [7].

The treatment strategies for MCI are divided into pharmacological and non-pharmacological interventions. Cholinesterase inhibitors, particularly donepezil, have been studied for MCI. Although some studies report symptomatic cognitive benefits, there is no established evidence that they modify disease progression, and treatment may be limited by adverse effects and uncertain long-term benefit [8, 9]. Non-pharmacological methods like cognitive training have certain prospects for reducing cognitive decline, but will encounter problems such as low patient compliance [10], insufficient caregiver support [11], and poor sustainability in clinical transformation [12]. Furthermore, given the multifactorial and complex pathological nature of neurodegenerative changes associated with MCI, monotherapies targeting a single pathway have inherent limitations in altering the long-term disease trajectory. In clinical practice, treatment regimens combining complementary therapies with medication are increasingly being adopted. In a meta-analysis of acupuncture therapy that included 24 RCTs (2005 patients), Sun et al. confirmed that the combination of acupuncture and oral medication yielded superior cognitive outcomes compared to oral medication alone (MoCA: MD = 0.52, *p* = 0.03; MMSE: MD = 0.91, *p* = 0.01) [13].

Acupuncture is a complementary approach that has attracted attention as a non-pharmacological adjunct in the management of MCI and Alzheimer’s disease (AD), owing to its potential cognitive and neurobiological effects and generally favorable safety profile [14, 15]. Previous meta-analyses suggest that acupuncture may improve short-term cognitive scale scores in patients with MCI; however, the certainty of evidence remains low or very low [15, 16]. In an updated systematic review, Cai et al. included 7 trials involving 536 participants and found that acupuncture, when used as an adjunct to conventional treatment, significantly improved MMSE scores (MD = 2.15, 95% CI: 1.61–2.69). In a meta-analysis incorporating 15 RCTs, Lin et al. confirmed that acupuncture-based therapies can lead to significant improvements in MMSE scores (MD = 1.09) [17, 18]. Although a growing body of clinical evidence suggests that acupuncture alone can effectively improve cognitive function in patients with MCI, the complexity of neurodegenerative disorders often makes it difficult for a single intervention to achieve optimal long-term outcomes. Currently used Western medications primarily target a single biochemical target; while they can provide short-term symptom relief, long-term use is often associated with gastrointestinal adverse reactions and diminishing efficacy. In contrast, acupuncture exerts multi-target regulatory effects, improving cerebral blood flow, inhibiting neuroinflammation, and promoting BDNF-related synaptic plasticity, providing a biologically plausible rationale for investigation in MCI [19, 20].

Consequently, the combined acupuncture and medication approach—which integrates the precise targeted action of medications with the systemic network regulation of acupuncture—is gaining increasing clinical attention. This combined strategy may hold promise for reducing medication dosage and adverse events while producing synergistic effects. Notably, a meta-analysis published by Bian et al., which searched seven databases and included 13 RCTs involving a total of 916 patients. The results showed that, compared with Western medicine alone, the combination of acupuncture and Western medicine significantly improved patients’ MMSE scores (MD = 1.79, 95% CI: 1.11–2.48), Montreal Cognitive Assessment (MoCA) scores (MD = 2.15, 95% CI: 1.47–2.83), and activities of daily living (ADL) scores [21]. A systematic review published by Ting et al. in 2016 was the first to propose this conclusion; it searched eight databases and included 18 RCTs, reporting that the combination of acupuncture and medication resulted in a combined increase of 1.73 points in MMSE scores [22]. Although the evidence base has gradually improved, existing studies still have significant methodological limitations: the included RCTs were generally published early, database coverage was incomplete, sample sizes were relatively small, and the versions of bias risk assessment tools were outdated; at the same time, the heterogeneity of the efficacy of acupuncture combined with medication across different MCI subtypes remains unclear; the differences in relative efficacy between acupuncture combined with Western medicine and acupuncture combined with traditional Chinese medicine have not yet been clarified; and the dose–response relationship between key acupuncture parameters—such as needle retention time and treatment duration—and the efficacy of the combined intervention requires further study.

Therefore, this study will conduct an updated systematic review and meta-analysis, incorporating all RCTs comparing “acupuncture combined with medication” with “medication alone” for the treatment of MCI. This review will strictly adhere to evidence-based methodology, including a comprehensive search of nine Chinese and English databases for literature published before July 2026, dual independent screening and data extraction, risk of bias assessment using RoB 2, and GRADE evidence grading. This study is expected to provide a basis for guiding the optimization of clinical decision-making. The specific research objectives are as follows:

1. Primary analysis: to investigate whether acupuncture combined with medication is more effective than medication alone.
2. Exploratory analysis: to explore the different efficacy of acupuncture in different MCI subtypes.
3. Exploratory analysis: to explore whether the synergistic effect varies with different drug categories.
4. Exploratory analysis: to explore the dose-response relationship between acupuncture parameters and combined treatment outcomes.

## Methods

This review and meta-analysis, as per the pre-developed protocol, will evaluate the efficacy of acupuncture combined with medication in mild cognitive impairment, namely MCI. Our proposal has been registered on the PROSPERO website under registration number CRD420261367845.

### Eligibility criteria

To ensure the selection process is both structured and transparent, the research eligibility criteria based on PICOS (Patient or Population, Intervention, Comparison, Outcome) will be listed [23].

#### Study design

##### Inclusion criteria

(1) randomized controlled trials (RCTs), (2) published in English or Chinese, (3) published by July 2026, (4) this study includes conference abstracts in the search and screening process to minimize the risk of publication bias. Conference abstracts will be retrieved and retained once sufficient data are obtained; if the criteria are not met, the authors will be contacted, and the study will be classified as “pending” rather than being directly excluded solely based on its publication format.

##### Exclusion criteria

(1) duplicate published studies, (2) studies with incomplete/unrecoverable data, (3) studies using animal models, (4) reviews, case reports or letters, (5) retracted articles.

#### Participants

##### Inclusion criteria

(1) participants have a formal diagnosis of MCI, based on many widely recognized criteria such as DSM-5, ICD-10, and NIA-AA. (2) Subjective or objectively reported cognitive decline. (3) Over 18 years old, have clear consciousness and stable vital signs. (4) Patients with severe neurological, psychiatric, or multiple organ dysfunctions (liver, kidney, cardiovascular) will be excluded if they have unstable severe neurological, psychiatric, or major organ dysfunction that could confound the cognitive assessment or increase the risk of bias.

Excluding confounding factors can prevent the results from being affected by unrelated systemic or mental health pathologies.

#### Interventions

The experimental group needs to receive acupuncture (including body acupuncture, scalp acupuncture, or electroacupuncture, without restrictions on acupoint selection) and also needs to receive drug therapy (such as acetylcholinesterase inhibitors, N-methyl-D-aspartate receptor antagonists or traditional Chinese medicine preparations). To ensure the reproducibility of the interventions, the included randomized controlled trials must clearly report the specific dosage parameters of acupuncture treatment, including needle retention time, treatment frequency, and total number of sessions. Trials in which acupuncture was the sole treatment method in the experimental group, or those that did not report the basic parameters of the acupuncture intervention, will be excluded. Efficacy data for Western medicines and traditional Chinese medicine formulations will be included in separate analysis groups for comprehensive evaluation.

#### Comparators

The control group received a single drug therapy with the same drug type and dose as the experimental group. To ensure the completeness of the analysis, it is also feasible if each experimental group simultaneously carries out common interventions such as routine nursing or cognitive rehabilitation.

#### Outcomes

Global cognitive function, as indexed by the Mini-Mental State Examination (MMSE) and the Montreal Cognitive Assessment (MoCA), will be the primary outcome [24]. Secondary outcomes include the Modified Barthel Index (MBI) [25] for function assessment, as well as the Rey Auditory Verbal Learning Test (RAVLT) [26] and the Digit Span Test [27] for quantifying memory and attention. In addition, to fully evaluate the clinical acceptability and safety of the combined intervention, the incidence, severity, and types of adverse events (AEs) reported during treatment will be systematically assessed as secondary outcomes.

### Search strategy

This study will conduct a comprehensive search across nine major databases, including PubMed, Embase, the Cochrane Library, Web of Science, China National Knowledge Infrastructure, Wanfang Data, the China Science and Technology Journal Database, the China Biomedical Literature Database, and Scopus. In addition, we will also search ClinicalTrials.gov, the World Health Organization’s International Clinical Trials Registry Platform, the China Clinical Trials Registry, as well as gray literature such as these, conference proceedings, reference lists from included studies, and relevant review articles.

Next, our search strategy will follow the PICO framework, use both Medical Subject Headings (MeSH) and free-text terms to conduct a comprehensive search across relevant fields in each database (Table 1). The specific search strategy for all databases is detailed in the Supplementary Materials.

**Table 1:** Search strategies for PubMed

Number	Terms
#1	(“Cognitive Dysfunction”[Mesh] OR “mild cognitive impairment”[tiab] OR “mild cognitive impairments”[tiab] OR “cognitive impairment, mild”[tiab] OR “amnestic mild cognitive impairment”[tiab] OR “non-amnestic mild cognitive impairment”[tiab] OR aMCI[tiab] OR naMCI[tiab] OR VMCI[tiab])
#2	(“Acupuncture Therapy”[Mesh] OR “Acupuncture”[Mesh] OR acupuncture[tiab] OR “manual acupuncture”[tiab] OR electroacupuncture[tiab] OR “electro-acupuncture”[tiab] OR “scalp acupuncture”[tiab] OR “body acupuncture”[tiab] OR “auricular acupuncture”[tiab])
#3	((randomized controlled trial[pt] OR controlled clinical trial[pt] OR randomized[tiab] OR randomised[tiab] OR placebo[tiab] OR randomly[tiab] OR trial[tiab] OR groups[tiab]) NOT (animals[mh] NOT humans[mh]))
#4	#1 AND #2 AND #3

### Data collection

#### Study selection

Note Express 3 is used for literature management and screening. Two reviewers will independently screen literature in a double-blind, two-stage manner. If there are differences, resolve them through reaching a consensus or by a third reviewer. After initially screening the titles or abstracts, conduct a strict evaluation of the full text to determine if it is finally qualified. The whole process is presented in Supplementary Table 1.

#### Data extraction

Two separate individuals will enter data into a preset standard Excel spreadsheet. The extracted data needs to be cross-checked to ensure accuracy and reduce extraction bias. The extracted variables include the following: study characteristics (first author, year of publication); trial design; baseline demographic data; specific intervention details (subtypes of mild cognitive impairment, drug categories, acupuncture methods and needle retention time per session, treatment frequency and total course of treatment, detailed records of adverse events); and pre- and post-intervention outcome measures. If a study reports results at multiple time points, the assessment conducted immediately after the end of treatment will be extracted as the primary endpoint. Follow-up data will be extracted separately to assess long-term effects (Table 2). For multi-arm trials employing multiple acupuncture protocols, we will combine relevant treatment arms into a single group for comparison with the control group, following the guidance of the Cochrane Handbook to avoid unit-of-analysis error [28]. If combining treatments is clinically inappropriate, we will select the intervention group that most closely aligns with standard clinical practice. Details are as follows:

**Table 2:** Data extraction form

Data item	Variables to be extracted
Study identification	Title, first author, year, country/region, journal or conference source, publication language, and report status
Trial design	Randomization method, allocation concealment, blinding, number of arms, analysis population, sample-size calculation, and trial registration
Participants	Diagnostic criteria, MCI subtype, sample size by group, age, sex, education, disease duration, baseline cognitive scores, and relevant comorbidities
Experimental intervention	Acupuncture type, acupoints, needle stimulation, retention time, session frequency, total sessions, treatment duration, practitioner information, drug name/class, dose, route, and frequency
Comparator and co-interventions	The same medication used in the experimental group, including type and dose; routine nursing, cognitive rehabilitation, or other co-interventions applied equally across groups
Primary outcomes	MMSE and MoCA: scale/version, assessment time point, group sample size, mean, standard deviation, and change-from-baseline data when reported
Secondary outcomes	Modified Barthel Index, RAVLT, Digit Span Test, and other prespecified functional, memory, or attention outcomes, with complete summary statistics

### Risk of bias assessment

The risk of bias assessment will be conducted through a double-blind, independent review process, in which two researchers will independently use the current Cochrane RoB 2 tool to assess the risk of bias for each relevant outcome measure. This tool classifies the risk of bias in a study into three categories: “low risk of bias,” “some concerns,” or “high risk of bias.” The tool is divided into five modules, including the following: bias arising from the randomization process, bias from deviation from the intended intervention, bias from missing outcome data, bias from outcome measurement, and bias from selective reporting of results. Subsequently, the researchers will cross-check their assessment results to ensure the accuracy and consistency of the assessment process. If the two reviewers disagree, a third-party expert will make the final determination [28].

### Statistical analysis

#### Handling missing data

For missing or unclear data, we will contact the authors. Where feasible, the standard deviation for missing data will be derived using the Cochrane method from standard errors, confidence intervals, *p*-values, or other reported statistics; if this is not possible, interpolated values from clinically comparable studies may be used in pre-specified sensitivity analyses. The primary analysis will utilize the available data, and studies will not be excluded entirely due to the incompleteness of a single outcome measure [28].

#### Meta-analysis

If the trials have sufficient clinical and methodological homogeneity, a meta-analysis will be conducted using Stata 17.0. For continuous outcomes, the mean difference (MD) will be used when all studies in the meta-analysis employ exactly the same measurement scale. When studies measure the same conceptual outcome but use different psychometric scales, standardized mean differences (SMDs) will be calculated. In all cases, 95% confidence intervals (CI) will be used to describe statistical precision. Statistical heterogeneity will be assessed using the *I*^2^ statistic; when heterogeneity is low (*I*^2^ < 50%), a fixed-effects model will be used. When heterogeneity is substantial (*I*^2^ ≥ 50%), a random-effects model will be used. To further investigate the clinical and methodological sources of heterogeneity, we will prioritize subgroup analyses and meta-regression (when there are at least 10 studies included for a single factor). If excessive and unexplained heterogeneity persists (*I*^2^ > 75%), we will forgo quantitative synthesis and instead use a structured descriptive analysis for summarization [28, 29].

#### Subgroup analysis

Contingent on data availability, subgroup analyses will investigate heterogeneity and refine treatment effect estimates across various conditions. Specifically, we will examine:

1. Compare the efficacy of combined interventions with monotherapy, focusing on subgroup differences between combined treatment and drug-only intervention.
2. Differences in acupuncture treatment duration: exploring the potential impact of acupuncture dose on the efficacy of combined therapy. To investigate possible sources of clinical heterogeneity, we will conduct subgroup analyses based on needle retention time, treatment frequency, and total treatment duration.
3. Differences in disease type: examine the response to combined acupuncture and medication in patients with amnestic mild cognitive impairment (aMCI) versus non-amnestic mild cognitive impairment (naMCI);
4. Differences in combined medications: compare whether there are differences in synergistic effects when acupuncture is combined with different classes of medications (e.g., cholinesterase inhibitors, glutamate receptor antagonists, and traditional Chinese medicine formulations). Western medicine and traditional Chinese medicine were analyzed separately.

All of the above subgroup analyses included at least three studies per category, and sufficient sample sizes were ensured in each case to avoid overinterpretation of subgroup results with insufficient statistical power.

#### Sensitivity analysis

To test the robustness of the main results, a leave-one-out sensitivity analysis [28] will be conducted. This process will exclude individual studies, especially small-sample studies, studies with methodological problems, or studies with high heterogeneity, to evaluate their disproportionate impact on the pooled effect size.

#### Publication bias

We will use funnel plots to assess potential reporting bias in the included studies (when the number of included studies is ≥ 10). Although funnel plots are subjective and lack objective numerical benchmarks, to rigorously assess publication bias, we will also validate our findings using Egger’s regression analysis as a supplementary quantitative tool [30, 31].

### Quality of evidence

To comprehensively assess the quality of the studies included in this review, two evaluators will independently use the GRADE (Grades of Recommendation, Assessment, Development, and Evaluation) grading system, which classifies evidence quality into four levels: very low, low, moderate, and high. The rating may be lowered accordingly if there are risks of bias, inconsistent results, indirect evidence, imprecise data, or publication bias. If two reviewers disagree, a third reviewer may be called upon to resolve the disagreement. This approach not only significantly reduces subjectivity and errors in the assessment process but also allows for an evaluation of the overall quality of the included studies. Consequently, it provides more comprehensive and objective evidence to support the conclusions of the meta-analysis [32].

### Patient and public involvement

Our study is based entirely on published data; therefore, direct involvement of patients or the public in the design, conduct, reporting, and dissemination strategies of this study is excluded.

## Discussion

MCI is the phase that connects the normal aging process with fully developed dementia. Most studies suggest that acupuncture has certain benefits in improving cognitive function [33]. Previous systematic reviews have already conducted extensive investigations into the efficacy of acupuncture for MCI and the combination of acupuncture and medication. In 2016, Shuai et al. included 18 randomized controlled trials involving a total of 1095 patients. The results showed that acupuncture combined with medication improved MMSE scores (MD = 1.73, 95% CI: 1.28–2.18) and ADL scores (MD = 5.63, 95% CI: 4.40–6.87) compared to medication alone [22]. More directly, a systematic review published by Bian et al. in 2026, which included 13 randomized controlled trials involving a total of 916 patients, found that acupuncture combined with Western medicine improved MMSE scores (MD = 1.79, 95% CI: 1.11–2.48), MoCA scores (MD = 2.15, 95% CI: 1.47–2.83), and ADL scores (MD = 5.32, 95% CI: 2.35–8.28) compared to Western medicine alone [21]. These studies suggest that combined interventions may offer benefits in terms of cognitive and activities of daily living functions; however, they all exhibit significant methodological limitations: the RCTs included were generally published early, database searches were incomplete, sample sizes were relatively small, versions of bias risk assessment tools were outdated, heterogeneity among MCI subtypes remains unclear, the relative differences in efficacy between acupuncture combined with Western medicine versus Chinese medicine are unclear, and the dose–response relationship between key acupuncture parameters and the efficacy of combined interventions has not yet been clarified.

The contribution of this review lies in updating the existing evidence and conducting a more rigorous methodological re-evaluation. While Bian et al.’s search was current as of September 2024 [21], this study aims to extend the search period to July 2026 and will search nine Chinese and English databases to include relevant randomized controlled trials published since then. Additionally, this study will use the RoB 2 tool to assess the risk of bias and the GRADE approach to evaluate the certainty of the evidence [28, 32].

Different subtypes of MCI may have distinct pathological bases and treatment responses. Amnestic MCI is more closely associated with the neurodegenerative changes seen in Alzheimer’s disease, whereas non-amnestic and vascular MCI may involve executive function, attention, or cerebrovascular pathways to a greater extent [34]; therefore, the effects of combined interventions may vary. At the same time, different drug classes target different mechanisms of action; when acupuncture is combined with cholinesterase inhibitors, glutamate receptor antagonists, drugs that improve cerebral circulation, or traditional Chinese medicine formulations, it may produce different effects. Therefore, investigating the subgroup effects of acupuncture in combination with different drug classes in specific MCI subtypes is of significant clinical importance. However, some subgroups may have insufficient research data, leading to low statistical power. For subgroups with too few studies, only descriptive summaries will be provided to avoid overinterpreting results with low statistical power.

Previous studies have shown that parameters such as the frequency of acupuncture, needle retention time, course of treatment, and acupuncture technique may also influence treatment outcomes [18]. Therefore, the analysis of acupuncture parameters in this study is primarily intended to explore potential parameter-effect associations and generate hypotheses for future research; causal dose–response relationships cannot be inferred solely from observational subgroup results. If the number of available studies and the completeness of parameter reporting are insufficient, a formal meta-regression will not be conducted; instead, the analysis will focus primarily on stratified descriptions and sensitivity analyses.

Furthermore, we must candidly acknowledge the methodological limitations inherent in acupuncture research. The physical nature of acupuncture makes strict double-blinding extremely difficult, which increases the risk of bias in RCTs. The acupuncture treatment process can elicit a strong placebo effect. Even when using sham acupuncture as a control, distinguishing the therapeutic effects of acupuncture from placebo responses remains a significant challenge [15]; the evidence is concentrated in Chinese-language journals, and the statistical analyses exhibit high heterogeneity; therefore, unless there is sufficient data to support formal interaction tests, the planned subgroup and dose analyses will remain exploratory; our meta-analysis will strictly adhere to the GRADE framework, downgrading the evidence affected by these serious methodological limitations to ensure that the final interpretation is cautious and transparent.

In summary, this meta-analysis will examine differences in the efficacy of acupuncture across various subtypes of MCI, different drug classes, and different acupuncture dosages, as well as assess clinical safety. It helps to identify potential factors influencing the combination of acupuncture and drug therapy. Ultimately, this study aims to provide evidence-based guidance for clinicians to facilitate the implementation of optimal combined intervention strategies in the early stages of the disease.

## Supplementary Information


Additional file 1. PRISMA-P 2015 checklist.Additional file 2. Planned study-selection flow diagram.Additional file 3: Table 1. Search strategies for all databases.Additional file 4. Data-extraction form.

## Data Availability

No datasets were generated or analysed during the current study.

## Abbreviations

MCI: Mild cognitive impairment
RCT: Randomized controlled trial
MMSE: Mini-Mental State Examination
MoCA: Montreal Cognitive Assessment
MBI: Modified Barthel Index
RAVLT: Rey Auditory Verbal Learning Test
ADL: Activities of daily living

## References

[1] Petersen RC. Mild cognitive impairment. Continuum. 2016;22(2):404–18. 10.1212/CON.0000000000000313. 27042901 PMC5390929

[2] Ataollahi Eshkoor S, Mun CY, Ng CK, Hamid TA. Mild cognitive impairment and its management in older people. CIA. 2015;687. 10.2147/CIA.S73922. PMC4401355 25914527

[3] Liss JL, Seleri Assunção S, Cummings J, Atri A, Geldmacher DS, Candela SF, et al. Practical recommendations for timely, accurate diagnosis of symptomatic Alzheimer’s disease (MCI and dementia) in primary care: a review and synthesis. J Intern Med. 2021;290(2):310–34. 10.1111/joim.13244. 33458891 PMC8359937

[4] Dunne RA, Aarsland D, O’Brien JT, Ballard C, Banerjee S, Fox NC, et al. Mild cognitive impairment: the Manchester consensus. Age Ageing. 2021;50(1):72–80. 10.1093/ageing/afaa228. 33197937 PMC7793599

[5] Salemme S, Lombardo FL, Lacorte E, Sciancalepore F, Remoli G, Bacigalupo I, et al. The prognosis of mild cognitive impairment: a systematic review and meta‐analysis. Alz Dem Diag Ass Dis Mo. 2025;17(1):e70074. 10.1002/dad2.70074. PMC11898010 40078377

[6] World Health Organization. World Health Organization [Fact sheet]. Dementia. 2026. Available from: https://www.who.int/news-room/fact-sheets/detail/dementia. Cited 2026 Aug 1.

[7] Ngandu T, Lehtisalo J, Solomon A, Levälahti E, Ahtiluoto S, Antikainen R, et al. A 2 year multidomain intervention of diet, exercise, cognitive training, and vascular risk monitoring versus control to prevent cognitive decline in at-risk elderly people (FINGER): a randomised controlled trial. Lancet. 2015;385(9984):2255–63. 10.1016/S0140-6736(15)60461-5. 25771249

[8] Matsunaga S, Fujishiro H, Takechi H. Efficacy and safety of cholinesterase inhibitors for mild cognitive impairment: a systematic review and meta-analysis. JAD. 2019;71(2):513–23. 10.3233/JAD-190546. 31424411

[9] Zhang X, Lian S, Zhang Y, Zhao Q. Efficacy and safety of donepezil for mild cognitive impairment: a systematic review and meta-analysis. Clin Neurol Neurosurg. 2022;213:107134. 10.1016/j.clineuro.2022.107134. 35078087

[10] He Z, Tian S, Singh A, Chakraborty S, Zhang S, Lustria MLA, et al. A machine-learning based approach for predicting older adults’ adherence to technology-based cognitive training. Inf Process Manag. 2022;59(5):103034. 10.1016/j.ipm.2022.103034. 35909793 PMC9337718

[11] Zhou C, Ai Y, Wang S, Yuan Y, Zhang A, Hu H, et al. Barriers and facilitators to participation in electronic health interventions in older adults with cognitive impairment: an umbrella review. BMC Geriatr. 2024;24(1):1037. 10.1186/s12877-024-05645-3. 39725926 PMC11670401

[12] Kulmala J, Rosenberg A, Ngandu T, Hemiö K, Tenkula T, Hyytiä A, et al. Facilitators and barriers to implementing lifestyle intervention programme to prevent cognitive decline. Eur J Public Health. 2021;31(4):816–22. 10.1093/eurpub/ckab087. 34448856 PMC8505000

[13] Sun B, Fu Q, Lau YYG, Zhan Y. Acupoint therapy for enhancing cognitive function in patients with mild cognitive impairment: a systematic review and meta-analysis. J Integr Complement Med. 2025:27683605251394592. 10.1177/27683605251394592. 41218823

[14] Yin Z, Zhou J, Xia M, Chen Z, Li Y, Zhang X, et al. Acupuncture on mild cognitive impairment: a systematic review of neuroimaging studies. Front Aging Neurosci. 2023;15:1007436. 10.3389/fnagi.2023.1007436. 36875696 PMC9975578

[15] Yin Z, Li Y, Jiang C, Xia M, Chen Z, Zhang X, et al. Acupuncture for mild cognitive impairment: a systematic review with meta-analysis and trial sequential analysis. Front Neurol. 2023;13:1091125. 10.3389/fneur.2022.1091125. 36686535 PMC9853885

[16] Li X, Lai L, Lu L, Yan L, Deng K, Li Z, et al. Comparative efficacy of acupuncture-related techniques for mild cognitive impairment: a Bayesian network analysis. Front Neurol. 2022;13: 942682. 10.3389/fneur.2022.942682. 36457861 PMC9706122

[17] Cai AQ, Hu LY, Wang YY, Dong S, Li B, Yang GY, et al. Acupuncture for vascular mild cognitive impairment: an updated systematic review and meta-analysis of randomized controlled trials. Syst Rev. 2026. 10.1186/s13643-026-03207-9. PMC13501624 42231492

[18] Lin G, Yie SLJ, Guo S, Li X, Xu L. Clinical evidence of acupuncture for amnestic mild cognitive impairment: a systematic review and meta-analysis of randomized controlled trials. Complement Ther Med. 2025;88:103114. 10.1016/j.ctim.2024.103114. 39617303

[19] Yu CC, Du YJ, Wang SQ, Liu LB, Shen F, Wang L, et al. Experimental evidence of the benefits of acupuncture for Alzheimer’s disease: an updated review. Front Neurosci. 2020;14:549772. 10.3389/fnins.2020.549772. 33408601 PMC7779610

[20] Wu L, Dong Y, Zhu C, Chen Y. Effect and mechanism of acupuncture on Alzheimer’s disease: a review. Front Aging Neurosci. 2023;15:1035376. 10.3389/fnagi.2023.1035376. 36936498 PMC10020224

[21] Bian X, Chen Z, Zhong L, Zhao X, Guo H, Du F, et al. Systematic review and meta-analysis of acupuncture combined with conventional western medicine in the treatment of mild cognitive impairment. Int J Geriatr. 2026;47(1):22–31.

[22] Shuai T, Zhang H, Yi L, Tian X, Zeng Z, Wang Y, et al. The effect of acupuncture plus drug versus drug alone on patients with mild cognitive impairment: a systematic review. Tradit Med Res. 2016;1(1):11–20. 10.53388/TMR201601003.

[23] Shamseer L, Moher D, Clarke M, Ghersi D, Liberati A, Petticrew M, et al. Preferred reporting items for systematic review and meta-analysis protocols (PRISMA-P) 2015: elaboration and explanation. BMJ. 2015;349(jan02 1):g7647–g7647. 10.1136/bmj.g7647. 25555855

[24] Nasreddine ZS, Phillips NA, Bédirian V, Charbonneau S, Whitehead V, Collin I, et al. The Montreal Cognitive Assessment, MoCA: a brief screening tool for mild cognitive impairment. J Am Geriatr Soc. 2005;53(4):695–9. 10.1111/j.1532-5415.2005.53221.x. 15817019

[25] Shah S, Vanclay F, Cooper B. Improving the sensitivity of the Barthel Index for stroke rehabilitation. J Clin Epidemiol. 1989;42(8):703–9. 10.1016/0895-4356(89)90065-6. 2760661

[26] Van Der Elst W, Van Boxtel MPJ, Van Breukelen GJP, Jolles J. Rey’s verbal learning test: normative data for 1855 healthy participants aged 24–81 years and the influence of age, sex, education, and mode of presentation. J Int Neuropsychol Soc. 2005;11(3):290–302. 10.1017/S1355617705050344. 15892905

[27] De Tollis M, De Simone MS, Perri R, Fadda L, Caltagirone C, Carlesimo GA. Verbal and spatial memory spans in mild cognitive impairment. Acta Neurol Scand. 2021;144(4):383–93. 10.1111/ane.13470. 33999426

[28] Higgins JPT, Thomas J, Chandler J, Cumpston M, Li T, Page MJ, editors. Cochrane handbook for systematic reviews of interventions. London: Cochrane; 2024.

[29] Higgins JPT, Thompson SG, Deeks JJ, Altman DG. Measuring inconsistency in meta-analyses. BMJ. 2003;327(7414):557–60. 10.1136/bmj.327.7414.557. 12958120 PMC192859

[30] Egger M, Smith GD, Schneider M, Minder C. Bias in meta-analysis detected by a simple, graphical test. BMJ. 1997;315(7109):629–34. 10.1136/bmj.315.7109.629. 9310563 PMC2127453

[31] Sterne JAC, Sutton AJ, Ioannidis JPA, Terrin N, Jones DR, Lau J, et al. Recommendations for examining and interpreting funnel plot asymmetry in meta-analyses of randomised controlled trials. BMJ. 2011;343(jul22 1):d4002–d4002. 10.1136/bmj.d4002. 21784880

[32] Guyatt GH, Oxman AD, Vist GE, Kunz R, Falck-Ytter Y, Alonso-Coello P, et al. GRADE: an emerging consensus on rating quality of evidence and strength of recommendations. BMJ. 2008;336(7650):924–6. 10.1136/bmj.39489.470347.AD. 18436948 PMC2335261

[33] Deng M, Wang XF. Acupuncture for amnestic mild cognitive impairment: a meta-analysis of randomised controlled trials. Acupunct Med. 2016;34(5):342–8. 10.1136/acupmed-2015-010989. 27491382

[34] Sanford AM. Mild cognitive impairment. Clin Geriatr Med. 2017;33(3):325–37. 10.1016/j.cger.2017.02.005. 28689566

