# Supplementary material for: Effectiveness of acupuncture combined with medication for mild cognitive impairment: a systematic review and meta-analysis protocol
Source: Syst Rev. 2026 Aug 22;15:238. doi: 10.1186/s13643-026-03306-7 (PMC13531846; doi:10.1186/s13643-026-03306-7)
Supplement: Supplementary file 3 [file 13643_2026_3306_MOESM3_ESM.docx]

**Supplementary Table 1: Search strategies for all databases**

*Search coverage: database inception to 31 July 2026. The electronic strategies were designed for sensitivity; the required comparison (acupuncture plus medication versus the same medication alone) and English/Chinese publication language will be confirmed during screening.*

**PubMed**

| **Number** | **Terms** |
| --- | --- |
| #1 | ("Cognitive Dysfunction"[Mesh] OR "mild cognitive impairment"[tiab] OR "mild cognitive impairments"[tiab] OR "cognitive impairment, mild"[tiab] OR "amnestic mild cognitive impairment"[tiab] OR "non-amnestic mild cognitive impairment"[tiab] OR aMCI[tiab] OR naMCI[tiab] OR VMCI[tiab]) |
| #2 | ("Acupuncture Therapy"[Mesh] OR "Acupuncture"[Mesh] OR acupuncture[tiab] OR "manual acupuncture"[tiab] OR electroacupuncture[tiab] OR "electro-acupuncture"[tiab] OR "scalp acupuncture"[tiab] OR "body acupuncture"[tiab] OR "auricular acupuncture"[tiab]) |
| #3 | ((randomized controlled trial[pt] OR controlled clinical trial[pt] OR randomized[tiab] OR randomised[tiab] OR placebo[tiab] OR randomly[tiab] OR trial[tiab] OR groups[tiab]) NOT (animals[mh] NOT humans[mh])) |
| #4 | #1 AND #2 AND #3 |

**Embase**

| **Number** | **Terms** |
| --- | --- |
| #1 | 'mild cognitive impairment'/exp OR 'mild cognitive impairment':ti,ab OR 'mild cognitive impairments':ti,ab OR 'cognitive impairment, mild':ti,ab OR 'amnestic mild cognitive impairment':ti,ab OR 'non-amnestic mild cognitive impairment':ti,ab OR amci:ti,ab OR namci:ti,ab OR vmci:ti,ab |
| #2 | 'acupuncture'/exp OR 'acupuncture therapy'/exp OR 'electroacupuncture'/exp OR acupuncture:ti,ab OR 'manual acupuncture':ti,ab OR electroacupuncture:ti,ab OR 'electro-acupuncture':ti,ab OR 'scalp acupuncture':ti,ab OR 'body acupuncture':ti,ab OR 'auricular acupuncture':ti,ab |
| #3 | 'drug therapy'/exp OR 'combination drug therapy'/exp OR medication*:ti,ab OR drug*:ti,ab OR pharmacotherap*:ti,ab OR 'pharmacological treatment':ti,ab OR donepezil:ti,ab OR memantine:ti,ab OR rivastigmine:ti,ab OR galantamine:ti,ab OR 'huperzine a':ti,ab OR nimodipine:ti,ab OR 'cholinesterase inhibitor':ti,ab OR 'acetylcholinesterase inhibitor':ti,ab OR 'nmda receptor antagonist':ti,ab OR 'chinese herbal medicine':ti,ab OR 'traditional chinese medicine':ti,ab OR 'chinese medicine preparation':ti,ab OR 'herbal medicine':ti,ab |
| #4 | 'randomized controlled trial'/exp OR 'controlled clinical trial'/exp OR 'randomization'/exp OR random*:ti,ab OR placebo*:ti,ab OR trial:ti,ab OR groups:ti,ab |
| #5 | #1 AND #2 AND #3 AND #4 |
| Limit | Coverage: database inception to 31 July 2026; no electronic language limit; English/Chinese eligibility applied during screening. |

**Cochrane Library**

| **Number** | **Terms** |
| --- | --- |
| #1 | MeSH descriptor: [Cognitive Dysfunction] explode all trees |
| #2 | ("mild cognitive impairment" OR "mild cognitive impairments" OR "cognitive impairment, mild" OR "amnestic mild cognitive impairment" OR "non-amnestic mild cognitive impairment" OR aMCI OR naMCI OR VMCI):ti,ab,kw |
| #3 | #1 OR #2 |
| #4 | MeSH descriptor: [Acupuncture Therapy] explode all trees |
| #5 | MeSH descriptor: [Acupuncture] explode all trees |
| #6 | (acupuncture OR "manual acupuncture" OR electroacupuncture OR "electro-acupuncture" OR "scalp acupuncture" OR "body acupuncture" OR "auricular acupuncture"):ti,ab,kw |
| #7 | #4 OR #5 OR #6 |
| #8 | MeSH descriptor: [Drug Therapy] explode all trees |
| #9 | (medication* OR drug* OR pharmacotherap* OR "pharmacological treatment" OR donepezil OR memantine OR rivastigmine OR galantamine OR "huperzine A" OR nimodipine OR "cholinesterase inhibitor*" OR "acetylcholinesterase inhibitor*" OR "NMDA receptor antagonist*" OR "Chinese herbal medicine" OR "traditional Chinese medicine" OR "Chinese medicine preparation*" OR "herbal medicine"):ti,ab,kw |
| #10 | #8 OR #9 |
| #11 | #3 AND #7 AND #10, in Trials |
| Limit | Coverage: database inception to 31 July 2026. No additional randomized-trial filter was applied in CENTRAL. |

**Web of Science**

| **Number** | **Terms** |
| --- | --- |
| #1 | TS=("mild cognitive impairment" OR "mild cognitive impairments" OR "cognitive impairment, mild" OR "amnestic mild cognitive impairment" OR "non-amnestic mild cognitive impairment" OR aMCI OR naMCI OR VMCI) |
| #2 | TS=(acupuncture OR "manual acupuncture" OR electroacupuncture OR "electro-acupuncture" OR "scalp acupuncture" OR "body acupuncture" OR "auricular acupuncture") |
| #3 | TS=(medication* OR drug* OR pharmacotherap* OR "pharmacological treatment" OR donepezil OR memantine OR rivastigmine OR galantamine OR "huperzine A" OR nimodipine OR "cholinesterase inhibitor*" OR "acetylcholinesterase inhibitor*" OR "NMDA receptor antagonist*" OR "Chinese herbal medicine" OR "traditional Chinese medicine" OR "Chinese medicine preparation*" OR "herbal medicine") |
| #4 | TS=(random* OR placebo* OR trial OR groups) |
| #5 | #1 AND #2 AND #3 AND #4 |
| Limit | Web of Science Core Collection; Timespan: All years; search cutoff: 31 July 2026. Records published after the cutoff will be excluded during screening. |

**Scopus**

| **Number** | **Terms** |
| --- | --- |
| #1 | TITLE-ABS-KEY("mild cognitive impairment" OR "mild cognitive impairments" OR "cognitive impairment, mild" OR "amnestic mild cognitive impairment" OR "non-amnestic mild cognitive impairment" OR aMCI OR naMCI OR VMCI) |
| #2 | TITLE-ABS-KEY(acupuncture OR "manual acupuncture" OR electroacupuncture OR "electro-acupuncture" OR "scalp acupuncture" OR "body acupuncture" OR "auricular acupuncture") |
| #3 | TITLE-ABS-KEY(medication* OR drug* OR pharmacotherap* OR "pharmacological treatment" OR donepezil OR memantine OR rivastigmine OR galantamine OR "huperzine A" OR nimodipine OR "cholinesterase inhibitor*" OR "acetylcholinesterase inhibitor*" OR "NMDA receptor antagonist*" OR "Chinese herbal medicine" OR "traditional Chinese medicine" OR "Chinese medicine preparation*" OR "herbal medicine") |
| #4 | TITLE-ABS-KEY(random* OR placebo* OR trial OR groups) |
| #5 | #1 AND #2 AND #3 AND #4 AND PUBYEAR < 2027 |
| Limit | Coverage: database inception through publication year 2026 (PUBYEAR < 2027). Records published after 31 July 2026 will be excluded during screening. English/Chinese eligibility will also be applied during screening. |

**中国知网**

SU=(轻度认知障碍 + 轻度认知功能障碍 + 遗忘型轻度认知障碍 + 非遗忘型轻度认知障碍 + 血管性轻度认知障碍 + MCI + aMCI + naMCI + VMCI) * SU=(针刺 + 针灸 + 手针 + 电针 + 头针 + 体针 + 耳针) * SU=(药物 + 药物治疗 + 西药 + 常规药物 + 多奈哌齐 + 美金刚 + 卡巴拉汀 + 加兰他敏 + 石杉碱甲 + 尼莫地平 + 胆碱酯酶抑制剂 + NMDA受体拮抗剂 + 中药 + 中医药 + 中成药 + 中药制剂) * SU=(随机 + 随机对照 + 随机对照试验 + 随机分组 + 随机分配 + 对照试验 + RCT)；检索范围：建库至2026年7月31日。

**中国生物医学数据库**

(("轻度认知障碍"[常用字段:智能] OR "轻度认知功能障碍"[常用字段:智能] OR "遗忘型轻度认知障碍"[常用字段:智能] OR "非遗忘型轻度认知障碍"[常用字段:智能] OR "血管性轻度认知障碍"[常用字段:智能] OR "MCI"[常用字段:智能] OR "aMCI"[常用字段:智能] OR "naMCI"[常用字段:智能] OR "VMCI"[常用字段:智能]) AND ("针刺"[常用字段:智能] OR "针灸"[常用字段:智能] OR "手针"[常用字段:智能] OR "电针"[常用字段:智能] OR "头针"[常用字段:智能] OR "体针"[常用字段:智能] OR "耳针"[常用字段:智能]) AND ("药物"[常用字段:智能] OR "药物治疗"[常用字段:智能] OR "西药"[常用字段:智能] OR "多奈哌齐"[常用字段:智能] OR "美金刚"[常用字段:智能] OR "卡巴拉汀"[常用字段:智能] OR "加兰他敏"[常用字段:智能] OR "石杉碱甲"[常用字段:智能] OR "尼莫地平"[常用字段:智能] OR "中药"[常用字段:智能] OR "中成药"[常用字段:智能] OR "中药制剂"[常用字段:智能]) AND ("随机"[常用字段:智能] OR "随机对照"[常用字段:智能] OR "随机对照试验"[常用字段:智能] OR "随机分组"[常用字段:智能] OR "随机分配"[常用字段:智能] OR "RCT"[常用字段:智能]))；检索范围：建库至2026年7月31日。

**维普数据库**

(摘要=(轻度认知障碍 OR 轻度认知功能障碍 OR 遗忘型轻度认知障碍 OR 非遗忘型轻度认知障碍 OR 血管性轻度认知障碍 OR MCI OR aMCI OR naMCI OR VMCI)) AND (摘要=(针刺 OR 针灸 OR 手针 OR 电针 OR 头针 OR 体针 OR 耳针)) AND (摘要=(药物 OR 药物治疗 OR 西药 OR 多奈哌齐 OR 美金刚 OR 卡巴拉汀 OR 加兰他敏 OR 石杉碱甲 OR 尼莫地平 OR 中药 OR 中成药 OR 中药制剂)) AND (摘要=(随机 OR 随机对照 OR 随机对照试验 OR 随机分组 OR 随机分配 OR RCT))；检索范围：建库至2026年7月31日。

**万方数据库**

主题:(轻度认知障碍 OR 轻度认知功能障碍 OR 遗忘型轻度认知障碍 OR 非遗忘型轻度认知障碍 OR 血管性轻度认知障碍 OR MCI OR aMCI OR naMCI OR VMCI) AND 主题:(针刺 OR 针灸 OR 手针 OR 电针 OR 头针 OR 体针 OR 耳针) AND 主题:(药物 OR 药物治疗 OR 西药 OR 多奈哌齐 OR 美金刚 OR 卡巴拉汀 OR 加兰他敏 OR 石杉碱甲 OR 尼莫地平 OR 中药 OR 中成药 OR 中药制剂) AND 主题:(随机 OR 随机对照 OR 随机对照试验 OR 随机分组 OR 随机分配 OR RCT)；检索范围：建库至2026年7月31日。

**Additional sources and trial registries**

| **Source** | **Search method** |
| --- | --- |
| ClinicalTrials.gov | Condition/disease: "mild cognitive impairment" OR MCI; Other terms: acupuncture OR electroacupuncture OR "scalp acupuncture"; Intervention/treatment terms: medication OR drug OR pharmacotherapy; Study type: Interventional studies; coverage: inception to 31 July 2026. |
| WHO ICTRP | ("mild cognitive impairment" OR MCI) AND (acupuncture OR electroacupuncture OR "scalp acupuncture") AND (medication OR drug OR pharmacotherapy); coverage: inception to 31 July 2026. |
| Chinese Clinical Trial Registry (ChiCTR) | (轻度认知障碍 OR 轻度认知功能障碍 OR MCI) AND (针刺 OR 针灸 OR 电针 OR 头针) AND (药物 OR 西药 OR 中药); coverage: inception to 31 July 2026. |
| Theses and conference proceedings | Search the dissertation and conference modules of CNKI and Wanfang using the corresponding Chinese strategy above; screen conference records retrieved through Embase, Web of Science and Scopus; contact authors when an abstract does not contain sufficient outcome data. |
| Backward and forward citation searching | Screen reference lists of all included studies and relevant systematic reviews; conduct forward-citation searching for included studies in Web of Science and Scopus. Apply a search cutoff of 31 July 2026. |
