# Supplementary material for: Effectiveness of acupuncture combined with medication for mild cognitive impairment: a systematic review and meta-analysis protocol
Source: Syst Rev. 2026 Aug 22;15:238. doi: 10.1186/s13643-026-03306-7 (PMC13531846; doi:10.1186/s13643-026-03306-7)
Supplement: Supplementary file 4 [file 13643_2026_3306_MOESM4_ESM.docx]

**Supplementary Table 2: Data-extraction form**

| **Data item** | **Variables to be extracted** |
| --- | --- |
| Study identification | Title, first author, year, country/region, journal or conference source, publication language, and report status. |
| Trial design | Randomization method, allocation concealment, blinding, number of arms, analysis population, sample-size calculation, and trial registration. |
| Participants | Diagnostic criteria, MCI subtype, sample size by group, age, sex, education, disease duration, baseline cognitive scores, and relevant comorbidities. |
| Experimental intervention | Acupuncture type, acupoints, needle stimulation, retention time, session frequency, total sessions, treatment duration, practitioner information, drug name/class, dose, route, and frequency. |
| Comparator and co-interventions | The same medication used in the experimental group, including type and dose; routine nursing, cognitive rehabilitation, or other co-interventions applied equally across groups. |
| Primary outcomes | MMSE and MoCA: scale/version, assessment time point, group sample size, mean, standard deviation, and change-from-baseline data when reported. |
| Secondary outcomes | Modified Barthel Index, RAVLT, Digit Span Test, and other prespecified functional, memory, or attention outcomes, with complete summary statistics. |
| Safety and acceptability | Adverse-event type, severity and incidence; serious adverse events; treatment discontinuation; adherence; withdrawals and reasons. |
| Missing and multi-arm data | Missing-data handling, information requested from authors, imputed statistics, and details needed to combine or otherwise handle multiple eligible arms. |
| Reporting and support | Funding source, conflicts of interest, ethics statement, trial registration, protocol availability, and reported deviations from the protocol. |
| Notes for risk-of-bias assessment | Information relevant to all five RoB 2 domains for each prespecified result/outcome and time point. |
