# Supplementary material for: Effectiveness of acupuncture combined with medication for mild cognitive impairment: a systematic review and meta-analysis protocol
Source: Syst Rev. 2026 Aug 22;15:238. doi: 10.1186/s13643-026-03306-7 (PMC13531846; doi:10.1186/s13643-026-03306-7)
Supplement: Supplementary file 6 [file 13643_2026_3306_MOESM2_ESM.docx]

**Supplementary Figure 1: Planned study-selection flow diagram**
